# Understanding frames: A UK survey of parents and professionals regarding the use of standing frames for children with cerebral palsy

**DOI:** 10.1111/cch.12505

**Published:** 2017-08-15

**Authors:** J. Goodwin, A. Colver, A. Basu, S. Crombie, D. Howel, J. R. Parr, E. McColl, N. Kolehmainen, A. Roberts, J. Lecouturier, J. Smith, K. Miller, J. Cadwgan

**Affiliations:** ^1^ Institute of Health and Society Newcastle University Newcastle Upon Tyne UK; ^2^ Institute of Neuroscience Newcastle University Newcastle upon Tyne UK; ^3^ Newcastle upon Tyne Hospitals NHS Foundation Trust Newcastle upon Tyne UK; ^4^ Sussex Community NHS Foundation Trust Chailey Clinical Services Brighton UK; ^5^ Robert Jones and Agnes Hunt Orthopaedic and District Hospital NHS Trust Oswestry UK; ^6^ Evelina London Children's Hospital Guys and St Thomas' NHS Foundation Trust (Kings Health Partners) UK London UK

**Keywords:** cerebral palsy, physiotherapy, standing frames

## Abstract

**Background:**

Standing frames are used for children with cerebral palsy (CP). They may improve body structure and function (e.g., reducing risk of hip subluxation, and improving bladder and bowel function), improving activity (e.g., motor abilities) and participation (e.g., interaction with peers), but there is little evidence that they do. We aimed to identify current UK standing frame practice for children with CP and to understand stakeholder views regarding their clinical benefits and challenges to use.

**Method:**

Three populations were sampled: clinicians prescribing standing frames for children with CP (n = 305), professionals (health and education) working with children with CP who use standing frames (n = 155), and parents of children with CP who have used standing frames (n = 91). Questionnaires were developed by the co‐applicant group and piloted with other professionals and parents of children with CP. They were distributed online via clinical and parent networks across the UK.

**Results:**

Prescribing practice was consistent, but achieving the prescribed use was not always possible. Respondents in all groups reported the perceived benefits of frames, which include many domains of the International Classification of Functioning Disability and Health for Children and Youth. Challenges of use are related to physical space and child‐reported pain.

**Conclusions:**

These survey findings provide information from key stakeholders regarding current UK standing frame practice.

## 1 INTRODUCTION

Cerebral palsy (CP) occurs in 2.5 per 1,000 live births. Abnormalities of tone, posture, and movement are associated with secondary musculoskeletal complications such as joint contractures, reduced bone mineral density (BMD), fractures, and hip dislocation, leading to pain and progressive disability (Shevell, Dagenais, Hall, and Repacq Consortium, 2009). In an effort to reduce the impact of these symptoms and maintain body structure, postural management strategies are widely used in children with CP. For children who are nonambulant (Gross Motor Function Classification System [GMFCS] IV or V; Palisano et al., 1997), consensus opinion supports standing frame use as part of postural management (Gericke, 2006). A standing frame is a rigid frame with a wide base. A child is positioned in the standing frame with variable support that may enable movement of the head, upper body, and upper limbs, thus potentially improving their function and participation. For the lower limbs, standing is usually passive (i.e., continuous, and stationary loading) but can be dynamic (i.e., simulating the forces applied during natural walking).

In keeping with the International Classification of Functioning, Disability and Health for Children and Youth (ICF‐CY; World Health Organization, 2007), the revised definition of CP (Rosenbaum et al., 2007) recognizes the importance of activity and participation, beyond purely body structure and function, for children with CP. Potential benefits of standing frame use relate to all aspects of the ICF‐CY framework. Regarding body structure and function, standing frames may reduce the risk of joint contractures, hip dysplasia, and scoliosis; improve BMD and gastrointestinal, bowel, and respiratory function; and reduce pain. Considering activity and participation, standing frames may enhance ability to stand independently, transfer, use one's upper body in play, and increase social and communicative participation. The use of standing frames may be more beneficial or challenging in different environments.

Standing frames may also have disadvantages. Children report pain and discomfort; families report increased demands on their time, reducing family and child participation (Bush et al., 2010). A practical issue is that frames are large and require storage. Further, education staff have described practical difficulties in using frames (Hutton and Coxon, 2011). Standing frames are expensive (typically costing £800 to £2,500) and require adaptation or replacement as a child grows. Frames require considerable therapist time to prescribe and monitor use. Despite these, standing frames are part of accepted practice in the UK in children with CP GMFCS IV and V. Professionals have opinions informed by clinical experience; however, there is little research evidence to confirm whether standing frames are beneficial or cause harm. Recent systematic reviews (Bush et al.*,* 2010; Fehlings et al., 2012; Paleg, Smith, and Glickman, 2013) have demonstrated limited and conflicting evidence for their benefit with respect to body structure and function. Although the most recent review (Paleg, Smith, and Glickman, 2013) claimed a positive effect on BMD, hip stability, and range of movement at the hip, knee, and ankle with variable duration of standing frame use, Fehlings et al. (2012) found no such evidence. A consensus statement acknowledged the limited evidence (Gericke, 2006) but still recommended that standing frames be used from age 12 months in children with CP and GMFCS IV and V (Gericke, 2006).

Clearly, there remains a research gap in terms of the value of standing frames throughout childhood (Ben‐Shlomo and Kuh, 2002). Therefore, we aimed to survey current UK standing frame practice and determine various stakeholders' perceptions of the benefits and challenges of standing frame use.

**Key messages**

- Standing frames are widely used as part of postural management for children with cerebral palsy, despite limited evidence of clinical efficacy.
- Professionals and parents of children with cerebral palsy are invested in using standing frames. They report a variety of benefits; however, they also recognize many challenges associated with standing frame use.
- Prescribing practice is consistent across the UK, but achieving the prescribed use is not always possible due to resources, environment, and child and family factors.

## 2 METHODS

### 2.1 Participants

Three populations in the UK were sampled for this study:

- Prescribing clinicians: Professionals such as physiotherapists prescribing standing frames for children with CP, *n* = 305.
- Nonprescribing professionals: Professionals such as paediatricians, orthopaedic surgeons, physiotherapists, and education staff who do not prescribe standing frames but work with children with CP who use them, *n* = 155.
- Parents: Parents or carers of children with CP who currently use or have used a standing frame, *n* = 91.

Figure S1 indicates participant flow through the study from responses received to responses included in the final analysis.

### 2.2 Measure

A questionnaire was devised following a literature review, and consultation with parents and child health professionals. Although all versions explored similar concepts, separate versions of the questionnaire were designed for the three participant populations to ensure the questions were relevant and used appropriate language.

The questions included the demographic characteristics of respondents and their experience and use of standing frames, the indications for prescription of standing frames, frame choice and prescribing practice, perceived benefits and difficulties associated with frame use, and differences between prescribed and actual use. Most questions offered fixed‐choice responses, though there were some opportunities for free‐text.

### 2.3 Procedure

The research was approved by the East Midlands—Nottingham 1 Research Ethics Committee (15/EM/0495). Recruitment was UK wide and took place between March and May 2016. The survey was hosted on SurveyMonkey™. E‐mail and web‐based flyers were sent to potential participants with a link to the appropriate version of the survey.

A convenience sample was approached. Prescribing clinicians and nonprescribing professionals were approached through relevant National Royal Colleges, professional bodies and their national newsletters, and UK Child Development Teams via the British Academy of Childhood Disability. Parents were approached via clinical services located in the North East, South East, and West Midlands of England. Parents were also approached through national parent organizations such as the National Network of Parent Carer Forums and other parent groups such as the Peninsula Cerebra Research Unit for Childhood Disability Research. In addition, social media were used to allow those interested to link to the study website (https://research.ncl.ac.uk/understandingframes/) via relevant Facebook pages (e.g., Cerebra) and the study's Twitter feed (@UnderstandFrame). A £10 voucher was offered to all who completed the questionnaire.

### 2.4 Analysis

Data analysis was descriptive, largely reporting percentages of respondents in each category for each question.

## 3 RESULTS

### 3.1 Participants

Table 1 outlines the respondent characteristics. Most prescribing clinicians and a large number of nonprescribing professionals were physiotherapists working in community settings. The majority had more than 10 years' experience and used a variety of standing frame types. Sixty‐five percent of parents had children who used only one type of standing frame that was assessed, fitted, and monitored by a physiotherapist.

**Table 1:** Characteristics of the two professional groups and the children whose parents responded

Professional group	Prescribing clinicians *N* (%)	Nonprescribing professionals *N* (%)	Children whose parents responded	*N* (%)
Occupation			Child's distribution of CP	
Physiotherapist	302 (99)	49 (31.6)	Whole body	72 (79.1)
Occupational therapist	1 (0.3)	39 (25.2)	Both sides of the body but legs more than arms	14 (15.4)
Paediatrician	0	29 (18.7)	One side of the body only	5 (5.5)
Classroom teacher or support teacher	0	15 (9.6)	Missing	0
Therapy assistant or technical instructor	1 (0.3)	11 (7.1)		
Other health professional	0	7 (4.5)		
Technician ‐ engineering background	0	3 (1.9)		
Orthopaedic surgeon	0	2 (1.3)		
Missing	1 (0.3)	0		
Current working environment			Child's school type	
Inpatients	34 (11.1)	32 (20.6)	Special school	68 (74.7)
Outpatients	153 (50.2)	77 (49.7)	Mainstream	29 (31.9)
Community—home	263 (86.2)	79 (51)	College (post 16—with additional or special provision)	5 (5.5)
Community—education centre (school/preschool)	279 (91.5)	107 (69)	Other	11 (12.1)
Other	1 (0.3)	6 (3.9)	Missing	4 (4.4)
Missing	4 (1.3)	2 (1.3)		
Years working with children who use standing frames^a^			Child's age	
More than 10 years	173 (56.7)	83 (53.5)	More than 10 years	46 (50.5)
6–10 years	59 (19.3)	32 (20.6)	6–10 years	25 (27.5)
2–5 years	44 (14.4)	24 (15.5)	2–5 years	14 (15.4)
Less than 2 years	25 (8.2)	14 (9)	Less than 2 years	1 (1.1)
Missing	4 (1.3)	2 (1.3)	Missing	5 (5.5)
Groups of children with whom the clinicians work			Child's estimated GMFCS level^b^	
GMFCS I	15 (4.9)	9 (5.8)	GMFCS I or II	8 (8.8)
GMFCS II	79 (25.9)	33 (21.3)	GMFCS III	20 (22)
GMFCS III	244 (80)	74 (47.7)	GMFCS III or IV	10 (11)
GMFCS IV	289 (94.8)	105 (67.7)	GMFCS IV	36 (39.6)
GMFCS V	277 (90.8)	95 (61.3)	GMFCS V	17 (18.7)
Would rely on prescriber	—	25 (16.1)	Missing	0
I am not familiar with GMFCS	5 (1.6)	12 (7.7)		
Missing	12 (3.9)	9 (5.8)		

*Note*.

a Percentages add up to greater than 100% because participants could choose more than one option.

b Although there is evidence that parents can accurately assess their child's Gross Motor Function Classification System (GMFCS) level (Morris, Galuppi, and Rosenbaum, 2004), feedback from parents during our preliminary engagement work indicated they did not want to be asked to categorize their child in this way. Therefore, we estimated the GMFCS level from reported information about independent walking, use of mobility aids, weight bearing, and maintenance of head position.

Children of the parent‐respondents were aged 1–18 years (median 10 years 6 months). They began standing frame use at 1–11 years (median 3 years) and stopped use at 3–16 years (median 9 years 7 months). Waiting times to receive a standing frame once recommended ranged between “less than 4 weeks” and “more than 26 weeks.”

### 3.2 Prescribing practice and actual use of standing frames

Standing frame recommendations and prescriptions for use were primarily based on clinical experience rather than on national or local guidance, as reported by both nonprescribing professionals and prescribing clinicians (81% and 89%, respectively).

Eighty‐two percent of prescribing clinicians suggested that standing frames should be used daily; however, only 18% of parents reported that this was achieved. Further, 76% of prescribers recommended the duration of standing should be 30–60 min, yet only 52% of parents reported this duration of use, with longer or shorter periods of use reported by 24% and 12% of participants, respectively (see Table 2).

**Table 2:** Prescribed and actual standing frame use

	Prescription of prescribing clinicians *N* (%)	Views of nonprescribing professionals *N* (%)	Parents: Prescribed use^a^ *N* (%)	Parents: Actual use^a^ *N* (%)
Frequency of use				
Everyday use	251 (82.3)	93 (60)	21 (31.3)	12 (17.9)
More than three times each week	38 (12.5)	15 (9.7)	29 (43.3)	26 (38.8)
More than once each week	0	0	5 (7.5)	9 (13.4)
Once each week	0	0	1 (1.5)	9 (13.4)
Less than once each week	0	0	0	3 (4.5)
I don't know	—	27 (17.4)	7 (10.4)	5 (7.5)
Missing	16 (5.2)	20 (12.9)	4 (6)	3 (4.5)
Duration of standing				
Less than 30 min	9 (3)	4 (2.6)	6 (9)	8 (11.9)
30 to 60 min	233 (76.4)	66 (42.6)	39 (58.2)	35 (52.2)
1 to 2 hr	46 (15.1)	18 (11.6)	11 (16.4)	16 (23.9)
More than 2 hr	1 (0.3)	1 (0.6)	0	0
I don't know	—	46 (29.7)	7 (10.4)	5 (7.5)
Missing	16 (5.2)	20 (12.9)	4 (6)	3 (4.5)

*Note*. Dash indicates that the item was not a response option for that group of participants.

a Percentages were calculated out of a total of 67, because this is how many participants were eligible to respond to those questions (parents who had a child who currently uses a standing frame).

The majority of prescribing clinicians suggested that standing frame use should be monitored and reviewed by the prescriber every 3 months or more often, but parents reported that monitoring and reviewing usually occurred every 3 months or less in practice.

### 3.3 Reasons for use, and perceived benefits and difficulties associated with standing frames

The prescribing clinicians and nonprescribing professionals who responded to the question about reasons for standing frame use in children with CP GMFCS IV and V consistently reported that they used the frames to offer the child a change of position; improve BMD, breathing, bladder, and bowel functions; reduce risk of fractures and joint contractures; reduce risk of hip dislocation or damage; and improve motor abilities, communication, vision, activity enjoyment, participation in activities, and peer interaction.

Parents reported the benefits they observed for their child (Table 3). Eighty‐nine percent of parents reported more than one benefit. When parents were asked to indicate the three most important benefits of standing frames, the most frequent choice was opportunity for a change of position; second was reduction of the risk of hip dislocation or damage, and equal third was improvement of bladder and bowel function, and reduced risk of joint contractures. Offering the child the opportunity for a change in position was also the most frequently reported indication of prescribing clinicians and nonprescribing professionals for standing frame use (Table 3).

**Table 3:** A comparison of professionals' rationale for prescribing, and parents' perceptions about the benefits

Benefits of standing frame use	Identified as an indication by prescribing clinicians^a^ *N* (%)	Identified as an indication by nonprescribing professionals^a^ *N* (%)	Parent reported benefits for their child^b^ *N* (%)
Opportunity for a change of position			
Less than 5 years of age	245 (80.3)	81 (52.3)	72 (79.1)
5 to 11 years of age	246 (80.7)	82 (51.6)	
12 to 18 years of age	244 (80)	81 (52.3)	
Participation in activities			
Less than 5 years of age	243 (79.7)	79 (51)	52 (57.1)
5 to 11 years of age	242 (79.3)	81 (52.3)	
12 to 18 years of age	238 (78)	79 (51)	
Enjoy activities			
Less than 5 years of age	231 (75.7)	77 (49.7)	39 (42.9)
5 to 11 years of age	230 (75.4)	79 (51)	
12 to 18 years of age	222 (72.8)	77 (49.7)	
Interaction with peers			
Less than 5 years of age	238 (78)	75 (48.4)	42 (46.2)
5 to 11 years of age	239 (78.4)	76 (49)	
12 to 18 years of age	233 (76.4)	73 (47.1)	
Reduce risk of joint contractures			
Less than 5 years of age	234 (76.7)	67 (43.2)	52 (57.1)
5 to 11 years of age	237 (77.7)	72 (46.5)	
12 to 18 years of age	232 (76.1)	69 (44.5)	
Improve bone density/strength			
Less than 5 years of age	217 (71.1)	70 (45.2)	56 (61.5)
5 to 11 years of age	224 (73.4)	71 (45.8)	
12 to 18 years of age	208 (68.2)	64 (41.3)	
Improve bladder and bowel functions			
Less than 5 years of age	225 (73.8)	66 (42.6)	52 (57.1)
5 to 11 years of age	231 (75.7)	69 (44.59)	
12 to 18 years of age	229 (75.1)	65 (41.9)	
Help child communicate			
Less than 5 years of age	217 (71.1)	68 (43.9)	12 (13.2)
5 to 11 years of age	217 (71.1)	67 (43.2)	
12 to 18 years of age	212 (69.5)	67 (43.2)	
Improve motor abilities (upper limbs)			
Less than 5 years of age	226 (74.1)	70 (45.2)	40 (44)
5 to 11 years of age	222 (72.8)	72 (46.5)	
12 to 18 years of age	201 (65.9)	62 (40)	
Improve motor abilities (head control)			
Less than 5 years of age	243 (79.7)	74 (47.7)	34 (37.4)
5 to 11 years of age	234 (76.7)	75 (48.4)	
12 to 18 years of age	196 (64.3)	64 (41.3)	
Reduce risk of hip dislocation or damage			
Less than 5 years of age	225 (73.8)	60 (38.7)	47 (51.6)
5 to 11 years of age	219 (71.8)	63 (40.6)	
12 to 18 years of age	195 (63.9)	56 (36.1)	
Improve breathing			
Less than 5 years of age	205 (67.2)	59 (38.1)	25 (27.5)
5 to 11 years of age	207 (67.9)	61 (39.4)	
12 to 18 years of age	208 (68.2)	60 (38.7)	
Help child use their vision			
Less than 5 years of age	173 (56.7)	58 (37.4)	21 (23.1)
5 to 11 years of age	170 (55.7)	56 (36.1)	
12 to 18 years of age	169 (55.4)	54 (34.8)	
Improve motor abilities (trunk control)			
Less than 5 years of age	221 (72.5)	60 (38.7)	45 (49.5)
5 to 11 years of age	217 (71.1)	62 (40)	
12 to 18 years of age	176 (57.7)	54 (34.8)	
Reduce risk of fractures			
Less than 5 years of age	175 (57.4)	47 (30.3)	23 (25.3)
5 to 11 years of age	175 (57.4)	51 (32.9)	
12 to 18 years of age	172 (56.4)	52 (33.5)	
Help child stand independently in future			
Less than 5 years of age	59 (19.3)	46 (29.7)	29 (31.9)
5 to 11 years of age	96 (31.5)	36 (23.2)	
12 to 18 years of age	55 (18)	23 (14.8)	
Help child walk in future			
Less than 5 years of age	120 (39.3)	29 (18.7)	17 (18.7)
5 to 11 years of age	77 (25.2)	22 (14.2)	
12 to 18 years of age	38 (12.5)	15 (9.7)	

a Responses of nonprescribing and prescribing clinicians refer to indications of standing frame use for Gross Motor Function Classification System (GMFCS) IV and/or V.

b Percentages add up to more than 100% because participants could choose more than one option.

Both prescribing clinicians and nonprescribing professionals reported that environmental and personal factors determined the most appropriate standing frame to use. They highlighted the issues of cost, space for use and storage, availability of frames, and parent/young person choice of frame.

Table 4 outlines the difficulties that prescribing clinicians, nonprescribing professionals, and parents experienced. Resourcing and environmental factors included funding for frames (87% of nonprescribing professionals), physical space in the home (78% of prescribing clinicians), and a child having a standing frame at nursery/school but not at home (55% of parents). Child factors as identified by the respondents included needing a rest from using a frame (25.3%), dislike of using a standing frame (19.8%), and experiencing pain (14.3%). These were more frequently reported by parents of children who no longer used frames (31.6% of parents of previous users reported pain compared with 10.4% of parents of current users.)

**Table 4:** Difficulties associated with standing prescription and use of standing frame

	Prescribing clinicians *N* (%)	Nonprescribing professionals *N* (%)	Parents (previous users and current users at home only)^a^ *N* (%)	Parents (current users but not at home)^b^ *N* (%)
Resources				
Allocation of resources or funding for frame	183 (60)	89 (87.4)	—	—
Allocation of resources for staff to prescribe/monitor use	64 (21)	42 (27.1)	—	—
Time	—	—	25 (48.1)	4 (12.1)
Do not have a standing frame at home	—	—	—	18 (54.6)
Using a standing frame at home was not recommended	—	—	—	2 (6.1)
Availability of parents/carers at home to help position the child	166 (54.4)	74 (47.7)	14 (26.9)	9 (27.3)
Availability of staff/carers in school to help position the child	176 (57.7)	72 (46.5)		—
Environment				
Physical space in home	238 (78)	96 (61.9)	19 (36.5)	16 (48.5)
Physical space at school	124 (40.7)	53 (34.2)		—
Transportation of equipment	106 (34.8)	55 (35.5)	—	—
Sometimes moving and handling difficulties at home for child	—	—	14 (26.9)	6 (18.2)
Difficulty with access to other equipment used to position child in the frame	—	—	10 (19.2)	3 (9.1)
Child factors	—	—		
Child dislikes standing in their frame	—	—	14 (26.9)	4 (12.1)
Child sometimes wants a rest from using the frame	—	—	19 (36.5)	4 (12.1)
Child experiences pain when standing in their frame	—	—	12 (23.1)	1 (3)
Other	62 (20.3)	26 (16.8)	7 (13.5)	6 (18.2)

*Note*. Percentages add up to greater than 100% because participants could choose more than one option. Dash indicates that the item was not a response option for that group of participants.

a Percentages were calculated out of a total of 52, because this is the number of participants who were eligible to respond to those questions AND provided an answer (parents who had a child who currently uses a standing frame [only outside of the home] did NOT answer this question).

b Percentages were calculated out of a total of 33, because this the number of participants who were eligible to respond to those questions AND provided an answer (ONLY parents who had a child who currently uses a standing frame [but do not use it at home] answered this question).

## 4 DISCUSSION

This paper reports a survey of standing frame use in the UK for the postural management of children age 1–18 years with CP, including perceived benefits and challenges. Factors influencing the choice of different types of standing frame prescribed included funding, environmental restrictions, personal preferences, and difficulties of the child and family. The consistency in dosage recommended by prescribers may have been influenced by Stuberg's (1992) recommendation for 5 days a week for 60 min. The prescribed dosage is often not achieved.

There were a range of clinical indications and benefits described by all stakeholders, with some interesting similarities and variations between groups. The opportunity for a change in position was reported most frequently by parents (79.1%) and prescribing clinicians (80%). For many other indications such as peer interaction, similar proportions of nonprescribing professionals (48.1%) and parents (46.2%) reported it as a benefit, in comparison with prescribing clinicians where a higher percentage reported it as an indication (77.6%.) Unfortunately this survey cannot explain why this is the case; however, the findings are important to note both clinically and for potential research with respect to delivery of care, and outcome measures to evaluate the prescribed regime.

Most participants described perceived benefits of standing frames in terms of body structure and function such as bladder or bowel functions; activity such as improved motor abilities; and participation such as interaction with peers. They also reported other benefits such as improvement in BMD and prevention of hip dislocation. They noted challenges related to environmental and personal factors such as physical space and the child's pain.

With respect to body structure and function, participants perceived benefits despite the lack of evidence in the literature. For example, 72.5% of prescribing clinicians reported a belief that frames improve bladder and bowel functions, yet we found only one single‐case study in a child with CP and chronic constipation (Rivi et al., 2014).

Respondents also perceived standing frames to help with participation, enjoyment, and communication. Physical assistance and environmental adaptations improve participation in children with CP (Schenker, Coster, and Parush, 2006), but there is no research relating specifically to standing frames. Being at standing height may be advantageous for social interaction and independence, but this is dependent on the position and activities of other individuals. When a person is using a wheelchair, a standing companion receives more eye contact from third parties, giving the impression that the wheelchair user depends on their standing companion (Edelmann et al., 1984). In terms of activity, upper limb function can be affected by positioning. Self‐feeding may be enhanced by standing, but picking up small objects is easier if sitting (Noronha, Bundy, and Groll, 1989). Therefore, it is necessary to explore how standing frames can promote or restrict participation in specific activities, at various times and different environments and in children of different ages.

Each participant group identified significant “environmental” challenges, particularly physical space for use and storage. Huang et al. also found space to be a major factor restricting assistive device use (including standing frames) by parents and teachers in their study in Taiwan (Huang, Sugden, and Beveridge, 2009a, 2009b). Other barriers in their study included inadequate teacher training, and personal factors such as feeling pressured to use equipment at school but not at home (Huang et al., 2009b). Huang did not report on carer availability for moving and handling, which was a reported difficulty in our sample.

### 4.1 Limitations

Due to the methods of survey dissemination, we could not calculate response rates from each participant group. Physiotherapists engaged well, but we received fewer responses from orthopaedic surgeons and paediatricians than anticipated. Additionally, survey responders may have had stronger views or more experience with standing frames than nonresponders, introducing bias. This survey sought views from professionals and parents and asked them to report their children's views. Although standing frame users' perspectives are essential, a survey is not an appropriate way to access children and young people with CP GMFCS IV and V.

Specific limitations of some questions became apparent during analysis. Prescribing clinicians and nonprescribing professionals were asked to answer questions with respect to children with CP GMFCS IV and V, whereas parents were asked about standing frames in relation to their own child: Some parents had children with better mobility. This may explain some of the differences between the perceptions of parents and clinicians. Further, there was no specific question regarding maintenance of range of movement for therapeutic reasons (e.g., stepping and standing to aid transfer) “versus” functional mobility (e.g., maintaining ambulation in adulthood).

## 5 CONCLUSIONS

This is the first survey of UK standing frame practice. It demonstrates investment of health professionals, education staff, and parents in the use of standing frames, who report a range of clinical indications and perceived benefits. It also provides insight into the challenges of use, which may impact on adherence to a prescribed standing programme. We present findings that provide a platform for considering (a) clinical delivery of the intervention, (b) assessment of appropriate outcomes according to the indications and perceived benefits, and (c) how we may develop further evaluative research regarding standing frame use in young people with CP.

## CONFLICT OF INTERESTS

The authors declare that the work submitted is their own and that copyright has not been breached in seeking its publication.

## Supporting information

Figure S1. Flow diagram of participants from responses received to responses in final analysis.
