# Supplementary figures and images for: Understanding frames: A UK survey of parents and professionals regarding the use of standing frames for children with cerebral palsy
Source: Child Care Health Dev. 2017 Aug 15;44(2):195–202. doi: 10.1111/cch.12505 (PMC5811781; doi:10.1111/cch.12505)

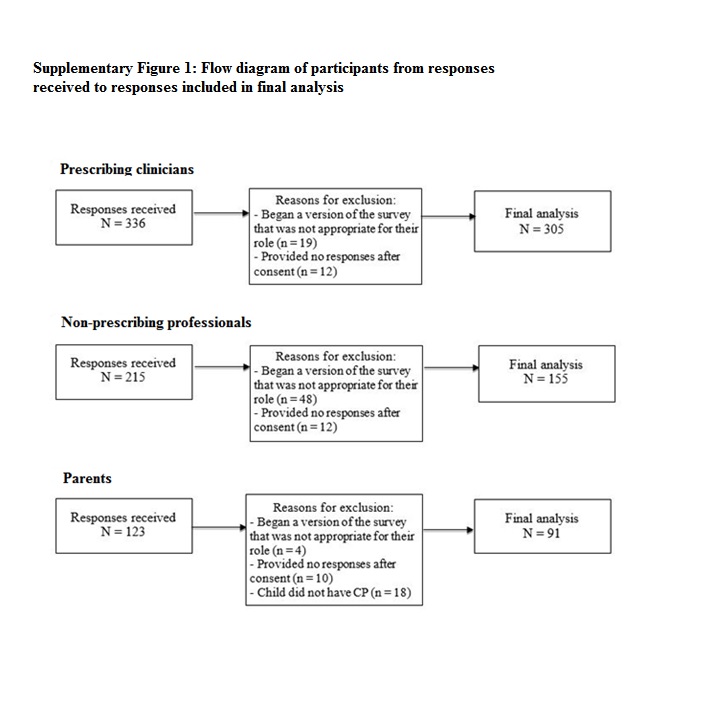

Supplement: Supplementary file 1 — Figure S1. Flow diagram of participants from responses received to responses in final analysis. [file CCH-44-195-s001.jpg]
